# Construction and application of mammary and breast cancer organoids

**DOI:** 10.1016/j.cellin.2026.100354

**Published:** 2026-08-17

**Authors:** Yuan Meng, Pinyang Bai, Lingxian Zhang, Mengna Zhang, Cheguo Cai

**Affiliations:** Key Laboratory of Systems Health Science of Zhejiang Province, School of Life Science, Hangzhou Institute for Advanced Study, University of Chinese Academy of Sciences, Hangzhou, Zhejiang, 310024, China

**Keywords:** Organoid, Mammary gland, Breast cancer, Application

## Abstract

Mammary organoids and breast cancer organoids (BCOs) have become indispensable models for investigating mammary development, tumorigenesis, therapeutic response, and drug resistance. In this review, we summarize current strategies for constructing mammary organoids from adult mammary stem cells, pluripotent stem cells and immortalized mammary epithelial cell lines, and discuss how these models recapitulate key physiological processes. Additionally, we elaborate on four complementary approaches for building BCOs, including patient-derived tumor organoids (PDOs), gene-edited models, microenvironment reconstruction via multicellular co-culture, and organoid-on-a-chip platforms. Notably, we outline major applications of mammary organoids in developmental biology and translational fields, and emphasize the expanding roles of BCOs in living biobanks, mechanistic studies, high-throughput drug screening, personalized drug sensitivity testing, and immunotherapy response prediction. Finally, we point out key challenges including standardization, long-term genetic and phenotypic stability, vascularization, immune-context integration, and optimization of dynamic culture conditions, and propose directions to improve model fidelity and clinical utility.

## 1 Introduction

The development of the mammary gland is a highly complex and finely regulated dynamic process, encompassing multiple stages including the embryonic period, puberty, pregnancy, and lactation period (Brisken & O'Malley, 2010; Hennighausen & Robinson, 2005; Macias & Hinck, 2012). Throughout this process, mammary epithelial cells undergo proliferation, differentiation, migration, and polarization, which are coordinately regulated by hormones, extracellular signaling pathways, and microenvironmental factors (Bach et al., 2017; Debnath et al., 2003; Ewald et al., 2008; Wiseman & Werb, 2002). However, dysregulation of this intricate regulatory process can lead to various breast diseases, including breast cancer (Yu et al., 2016; Zhang et al., 2022), mastitis (Amir et al., 2007; Kvist et al., 2008; Santen et al., 1990), breast fibrosis (Boyd et al., 2007; Guray & Sahin, 2006), and fibroadenomas (El-Wakeel & Umpleby, 2003; Guray & Sahin, 2006), with breast cancer being the most severe and clinically impactful.

Breast cancer remains the most commonly diagnosed malignancy in women worldwide. It continues to pose a major public health burden with an estimated 2.309 million new cases and 665 700 deaths in 2022 according to the latest data (Bray et al., 2024). Breast cancer is a heterogeneous disease, commonly classified into four main subtypes: Luminal A, Luminal B, HER2-positive (HER2^+^), and triple-negative breast cancer (TNBC) which differ markedly in prognosis and treatment response (Ellis & Perou, 2013; Visvader, 2011), and this heterogeneity complicates accurate diagnosis and effective treatment (Perou et al., 2000; Sørlie et al., 2001). Therefore, developing novel experimental models that faithfully recapitulate the characteristics of breast cancer and are suitable for mechanistic studies and therapeutic development is of paramount importance.

**Fig. 1:**
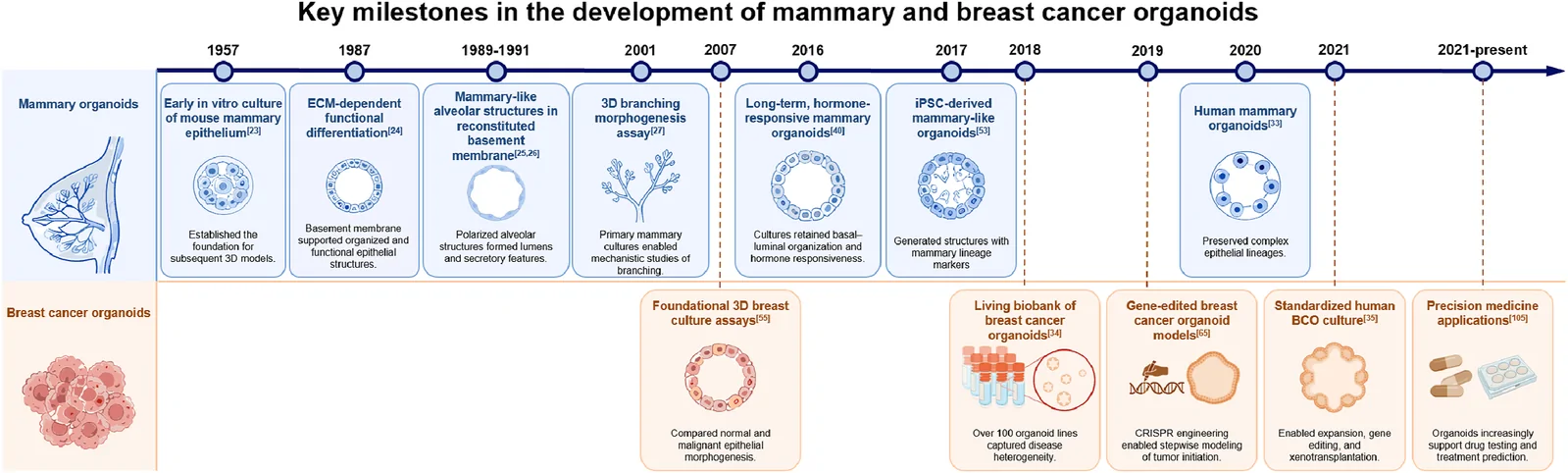
**Key milestones in the development of mammary and breast cancer organoids.** The upper timeline summarizes representative advances in mammary organoid research, including early *in vitro* culture of adult mouse mammary epithelium in 1957, extracellular matrix-dependent functional differentiation in 1987, formation of polarized mammary-like alveolar structures in reconstituted basement membrane during 1989–1991, establishment of three-dimensional branching morphogenesis assays in 2001, development of long-term hormone-responsive mammary organoids in 2016, generation of induced pluripotent stem cell-derived mammary-like organoids in 2017, and establishment of human mammary organoids preserving complex epithelial lineages in 2020. The lower timeline highlights major developments in breast cancer organoid research, including foundational three-dimensional breast culture assays in 2007, establishment of living breast cancer organoid biobanks in 2018, generation of gene-edited breast cancer organoid models in 2019, development of standardized human breast cancer organoid culture workflows in 2021, and the expanding use of these models in precision medicine from 2021 onward. Representative milestones are shown, and the timeline is not intended to be exhaustive. **BCOs**, breast cancer organoids; **ECM**, extracellular matrix; **iPSCs**, induced pluripotent stem cells.

The advent of organoid technology has provided an unprecedented platform for breast biology and breast cancer research. Organoids are miniature organ-like structures self-organized by stem cells or organ progenitor cells under *in vitro* three-dimensional culture conditions, which can largely recapitulate the physiological structure, cellular diversity, and specific functions of the source organ (Rossi et al., 2018). The establishment of long-term intestinal organoid culture in 2009 provided a general paradigm for modern epithelial organoid systems (Sato et al., 2009). However, mammary-specific *in vitro* culture and extracellular matrix-supported three-dimensional morphogenesis studies had already developed over several preceding decades. Building on these foundations, mammary and breast cancer organoid technologies have advanced toward long-term expandable cultures, living biobanks, gene-edited models, and precision medicine applications, with key milestones summarized in Fig. 1.

This review summarizes recent advances in the construction and application of mammary organoids and breast cancer organoids (BCOs). We first introduce the construction strategies and technical advances in organoid construction, including the culture systems for both mammary organoids and BCOs. Then we discuss in detail the applications of these models in basic research, translational medicine, toxicology, and synthetic biology. Finally, the challenges faced in this field will be discussed, along with prospects for future research.

## 2 Construction of mammary organoids

**Fig. 2:**
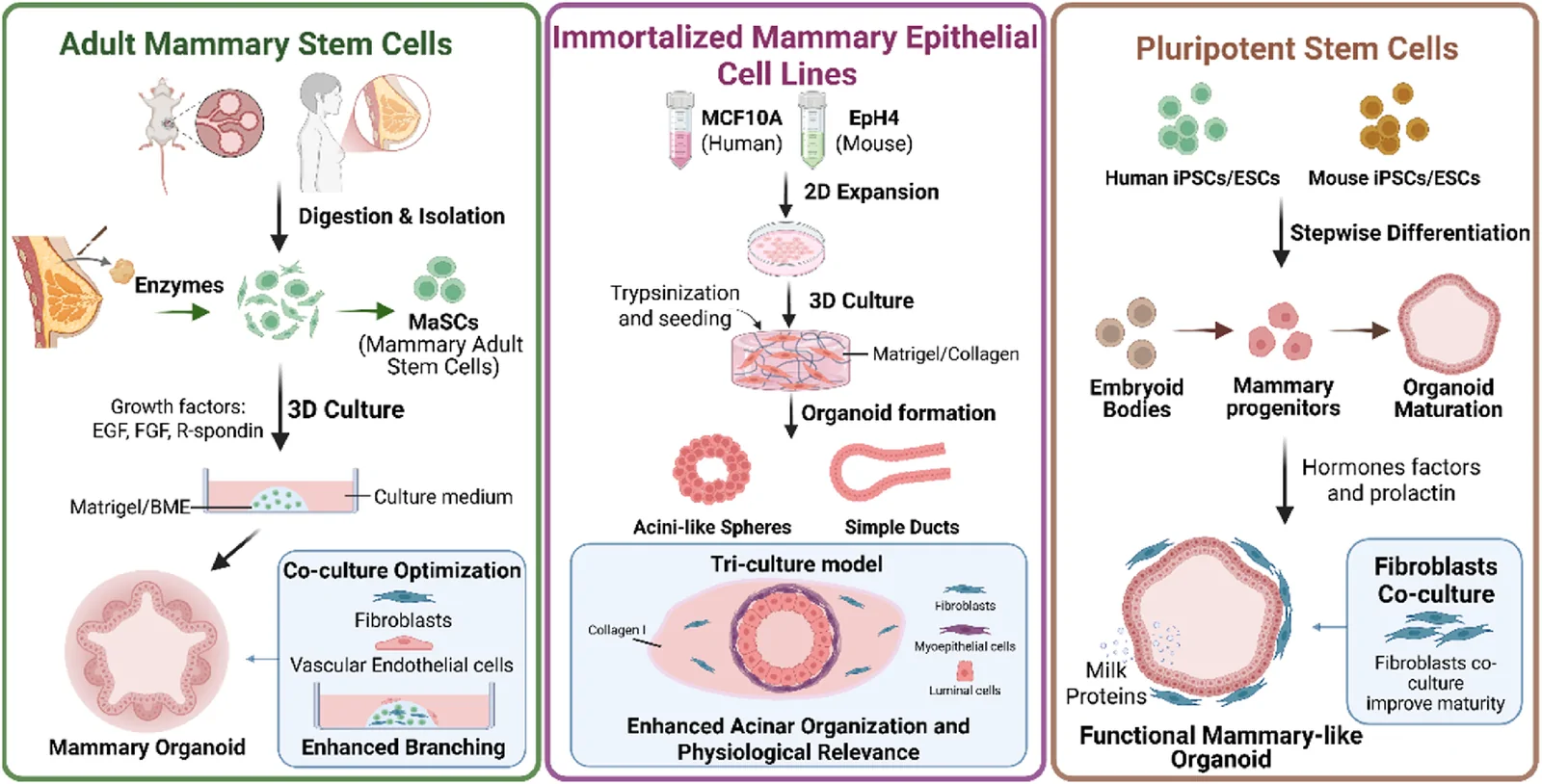
**Construction of mammary organoids from adult mammary stem cells, immortalized mammary epithelial cell lines, and pluripotent stem cells. Left:** Primary mammary epithelial or stem cells are isolated from mouse or human mammary tissues by mechanical and enzymatic dissociation and embedded in Matrigel or basement membrane extract for three-dimensional culture in growth factor-supplemented medium. Co-culture with fibroblasts and vascular endothelial cells can enhance ductal elongation and branching. **Middle:** Immortalized mammary epithelial cell lines, including human MCF10A and mouse EpH4 cells, are expanded in two-dimensional culture and subsequently embedded in Matrigel or collagen to generate acinar-like spheres or duct-like structures. Incorporation of luminal epithelial cells, myoepithelial cells, fibroblasts, and collagen I in multicellular culture systems improves acinar organization and physiological relevance. **Right:** Human or mouse pluripotent stem cells are subjected to stepwise differentiation through embryoid-body and mammary-progenitor stages to generate mammary-like organoids. Hormonal stimulation, including prolactin treatment, promotes functional maturation and milk-protein production, whereas fibroblast co-culture further improves structural maturity. BME, basement membrane extract; **EGF**, epidermal growth factor; **ESCs**, embryonic stem cells; **FGF**, fibroblast growth factor; **iPSCs**, induced pluripotent stem cells; **MaSCs**, mammary stem cells; **PSCs**, pluripotent stem cells.

Mammary organoids are three-dimensional models that recapitulate key aspects of mammary tissue structure and function. In 1957, Lasfargues established an *in vitro* culture system for adult mouse mammary epithelium (Lasfargues, 1957). In 1987, Li et al. demonstrated that reconstituted basement membrane supported mammary-specific functional differentiation (Li et al., 1987). Subsequent studies in 1989 and 1991 showed that primary mammary epithelial cells formed polarized alveolar-like structures with lumens and secretory features in reconstituted basement membrane (Aggeler et al., 1991; Barcellos-Hoff et al., 1989). Since then, multiple platforms have been established, which can be classified by cell source: adult mammary stem cells, immortalized mammary epithelial cell lines, and pluripotent stem cells (Fig. 2).

### 2.1 Organoids from adult mammary stem cells (MaSCs)

Primary mammary epithelial cells isolated from fresh mammary tissue better preserve diversity and biological features of the original mammary epithelium *in vitro* than traditional cell lines. MaSCs constitute a distinct epithelial population with self-renewal capacity and multilineage differentiation potential. The key research progress on mammary organoids derived from MaSCs is summarized in Table 1.

**Table 1:** Mammary organoids derived from adult mammary stem cells.

Species	Source of cells	Construction method	Key features	Application	Year	Ref.
Mouse	Primary mammary epithelial cells	EHS extract embedment	Ductal and luminal structures; milk protein secretion	ECM signaling	1987	Li et al. (1987)
Mouse	Primary mammary epithelial cells	Collagen gel + MMP/growth factors	Branching morphogenesis	Branching morphogenesis	2001	Simian et al. (2001)
Human	Normal and malignant mammary epithelial cells	EHS extract embedment	Simple epithelial colonies	Acinar morphogenesis; normal–tumor growth control comparison	2007	Lee et al. (2007)
Mouse	Primary mammary epithelial cells	Mammosphere + Notch modulators	Notch-dependent self-renewal and luminal fate	Lineage fate; stem cell regulation	2008	Bouras et al. (2008)
Mouse	Primary mammary epithelial cells	Matrigel embedment	Collective migration; ductal branching	Branching morphogenesis; lineage fate; tumorigenesis	2008	Ewald et al. (2008)
Mouse	Primary mammary epithelial cells	Matrigel/Collagen I mix	ECM-dependent branching and lumen formation	Branching morphogenesis; tumorigenesis	2015	Nguyen-Ngoc et al. (2015)
Human	Human normal mammary tissue	Matrigel dome + optimized medium	Differentiates into basal/luminal/ER^+^ cells	Lineage fate	2020	Rosenbluth et al. (2020)
Mouse	Primary mammary epithelial cells	Matrigel + FGF2/Prolactin cycling	Lactation and involution remodeling	Drug safety evaluation; hormonal regulation	2020	Sumbal et al. (2020)
Human	Primary human basal mammary epithelial cells	Floating collagen type I culture	ECM-guided ductal branching	Branching morphogenesis; mammary gland morphology	2021	Buchmann et al. (2021)
Human	Human normal and breast cancer tissues	BME embedment + optimized medium	Long-term expansion; retains subtype & mutation profiles	Long-term culture	2021	Dekkers et al. (2021)
Mouse	Mammary stem cells	Matrigel + co-culture (endothelial/fibroblast)	Long ductal structures	Branching morphogenesis; microenvironmental signaling	2021	Wang et al. (2021)
Mouse	Primary mammary epithelial cells	BME/Collagen I + alternating FGF2/EGF	Growth factor-driven ductal branching	Branching morphogenesis	2022	Caruso et al. (2022)
Human	Primary human basal mammary epithelial cells	Floating collagen	Oscillatory/rotational migration during branching	Branching morphogenesis	2022	Hutterer et al. (2022)
Mouse	Primary mammary epithelial cells	Matrigel + EGF/FGF	Growth factor-dependent expansion; bilayered ducts	Hormonal regulation; branching morphogenesis	2022	Sahu et al. (2022)
Human	Primary human mammary epithelial cells	Matrigel embedment	Basal/luminal layers; enhanced branching	Toxicology research; mammary gland morphology	2022	Winkler et al. (2022)
Mouse	Mammary stem cells (Krt14^+^)	Matrigel + co-culture (trophoblasts)	Recapitulates puberty to lactation; hormone-responsive	Lineage fate; hormonal regulation	2023	Yuan et al. (2023)
Mouse	Mammary stem cells	Matrigel + co-culture (macrophages)	Ductal features; hormone response; milk secretion	Epithelial-macrophage crosstalk	2023	Zhou et al. (2023)
Mouse	Primary mammary epithelial cells	Matrigel + Notch activation	Basal-to-luminal lineage conversion	Lineage fate; stem cell regulation	2025	Merle et al. (2025)

#### 2.1.1 Foundational studies in mouse models

Early work established foundational principles of mammary organoid formation from primary mouse cells. Embedding primary mammary epithelial cells in EHS extract or collagen gels supports ductal and luminal structure formation, with branching morphogenesis regulated by matrix metalloproteinases and growth factors (Li et al., 1987; Simian et al., 2001). Subsequent refinement using Matrigel and defined growth factors (e.g., EGF, FGF) enabled long-term maintenance and mechanistic studies of ductal morphogenesis (Ewald et al., 2008; Nguyen-Ngoc et al., 2015; Sahu et al., 2022).

#### 2.1.2 Advances in mouse organoid complexity through co-culture

To better recapitulate the *in vivo* mammary microenvironment, co-culture systems incorporating stromal, vascular, and immune cells have been developed. Co-culture of mammary epithelial or stem cells with endothelial cells and fibroblasts promotes ductal elongation and branching. Mechanistically, mammary fibroblasts relay Wnt signals derived from the endothelial niche to organize epithelial patterning and branching morphogenesis (Wang et al., 2021). Macrophages not only enhance ductal morphogenesis and hormonal responsiveness but also maintain mammary stem cell activity and mammary tissue homeostasis. Mechanistically, macrophage-derived TNF-α activates PI3K signaling and promotes Cdk1/Cyclin B1-dependent cell-cycle progression in mammary stem cells (Zhou et al., 2023). In macrophage-containing mammary organoids, prolactin stimulation further induces lactogenic differentiation and milk protein secretion (Zhou et al., 2023). Co-culture with trophoblasts recapitulates mammary developmental stages from embryogenesis to lactation and captures the transition from multipotent to unipotent mammary stem cells (Yuan et al., 2023).

#### 2.1.3 Establishment and optimization of human mammary organoid models

Long-term expandable human mammary organoid cultures were established in 2018 using optimized media containing EGF, R-spondin, Noggin, and Neuregulin-1, which support both basal and luminal lineages, including estrogen receptor-positive cells (Dekkers et al., 2021; Rosenbluth et al., 2020; Sachs et al., 2018). EGF binds to EGFR and activates downstream mitogenic signaling, including PI3K signaling, thereby supporting epithelial cell growth and organoid expansion (Jung et al., 2019). R-spondin-1 potentiates canonical Wnt/β-catenin signaling and promotes mammary stem cell self-renewal (Cai et al., 2014). In addition, R-spondin-1 regulates ERα expression through a Wnt/β-catenin-independent cAMP–PKA signaling pathway (Geng et al., 2020). Noggin functions as an extracellular BMP antagonist and contributes to the maintenance of a signaling environment permissive for mammary stem/progenitor cell expansion (Walsh et al., 2010). Neuregulin-1 activates ErbB-associated signaling and enables the prolonged expansion of mammary organoids while preserving their basal–luminal bilayered organization and responsiveness to steroid hormones (Jardé et al., 2016). Together, these factors maintain lineage diversity and functional characteristics during long-term organoid culture. Nevertheless, limited access to fresh human tissue and donor-to-donor variability remain important constraints.

### 2.2 Organoids from immortalized mammary epithelial cell lines

Compared to the previous approach, immortalized mammary epithelial cell lines have become an important tool for constructing controllable mammary organoid models due to their broad availability, ease of culture, and defined genetic background. The key research progress on mammary organoids derived from cell lines is summarized in Table 2.

**Table 2:** Mammary organoids derived from immortalized mammary epithelial cell lines.

Species	Source of cells	Construction method	Key features	Application	Year	Ref.
Mouse	EpH4 cell line	Collagen or Matrigel embedment	Matrix-dependent: branching ducts (collagen) vs. hollow cysts (Matrigel)	Branching morphogenesis; tumorigenesis	1998	Montesano et al. (1998)
Human	D492 cell line	Reconstituted basement membrane + co-culture (endothelial cells)	Endothelial-induced EMT; cadherin switch	EMT modeling; tumorigenesis	2011	Sigurdsson et al. (2011)
Human	HMT-3522 S1 cell line, MCF-10A cell line HB2 + myoepithelial/fibroblast	Matrigel + differentiation media	Apical junctions; lumen-containing breast-like architecture	Microenvironmental signaling study; tumorigenesis; acinar morphogenesis	2013	Vidi et al. (2013)
Human		Collagen type I culture + co-culture (myoepithelial cell line Myo1089 and fibroblasts)	Polarized duct-like structures; hollow lumens; BM deposition	Tumorigenesis	2015	Nash et al. (2015)
Human	D492 cell line	Reconstituted basement membrane embedment	Branching ducts; bipotent differentiation; EMT/MET capacity	EMT modeling; tumorigenesis	2019	Briem et al. (2019)
Human	MCF10A cell line	Scaffold-free, non-adhesive culture	Hollow lumens; co-expresses basal/luminal markers; TDLU mimic	EMT modeling; tumorigenesis	2019	Djomehri et al. (2019)
Human	HMT-3522 S1 cell line	Scaffold-free spheroid aggregation	Multilayered spheroids; asymmetric lumina	UDH recapitulation; lineage fate	2019	Florian et al. (2019)
Human	MCF10A cell line Comma-1D cell line	Hanging drop + minimal Matrigel	Basal-in phenotype; interior basement membrane	Drug screening, tumorigenesis	2021	Parigoris et al. (2021)
Mouse		Matrigel embedment	Heterogeneous organoids with luminal/basal/bipotent cells	Lineage fate; tumorigenesis	2022	Werner et al. (2022)

#### 2.2.1 Mammary organoids from mouse immortalized cell lines

Mouse cell lines such as Comma-1D and EpH4 have been used to study heterogeneity and matrix-dependent morphogenesis. Comma-1D-derived organoids allow dissection of epithelial heterogeneity and HER2-driven tumorigenesis (Danielson et al., 1984; Werner et al., 2022). EpH4 exhibits context-dependent morphogenesis, forming branching ducts in collagen and hollow cysts in Matrigel (Montesano et al., 1998).

#### 2.2.2 Mammary organoids from human immortalized cell lines

MCF10A cells form hollow acini in Matrigel, recapitulating terminal ductal lobular unit architecture through processes dependent on apoptotic signaling and E-cadherin-mediated polarization (Djomehri et al., 2019; Parigoris et al., 2021; Soule et al., 1990; Vidi et al., 2013).

Other human cell lines offer niche applications: HMT-3522 S1 models usual ductal hyperplasia (Florian et al., 2019). D492 co-cultured with endothelial cells enables study of epithelial-to-mesenchymal transition (EMT) in basal-like breast cancer (Briem et al., 2019; Sigurdsson et al., 2011) and HB2 forms acinar structures in collagen gels (Nash et al., 2015). While scalable and reproducible, these models lack the full complexity and hormonal responsiveness of primary tissue.

### 2.3 Mammary organoids from pluripotent stem cells

Although significant progress has been made in the development of mammary organoid models derived from primary cells and immortalized cell lines, their applications are still limited by challenges such as the restricted availability of cell sources, donor heterogeneity, and difficulties in simulating early developmental processes. Pluripotent stem cells (PSCs), including embryonic stem cells (ESCs) and induced pluripotent stem cells (iPSCs), possess the potential for self-renewal and differentiation into all cell types of the three germ layers. This ability enables the *in vitro* reproduction of the mammary development process, facilitating the generation of mammary epithelial cell lineages from a non-structured state and providing a novel breakthrough in mammary organoid research (Takahashi & Yamanaka, 2006). The key research progress on mammary organoids derived from PSCs is summarized in Table 3.

**Table 3:** Mammary organoids derived from pluripotent stem cells.

Species	Source of cells	Construction method	Key features	Application	Year	Ref.
Human	Human induced pluripotent stem cells (hiPSCs)	Two-step: non-neural ectoderm enrichment → 3D floating mixed gel	Expresses luminal/basal/ER markers; milk protein production	Lineage fate transitions, stem cell regulation, drug screening	2017	Qu et al. (2017)
		Two-step differentiation + co-culture/add HFF	Increased branching, size, and multilayered duct-like structures	Cellular reprogramming, tissue engineering	2022	Dai et al. (2022)
Mouse	Mouse embryonic stem cells (mESCs)	Suspension 3D culture + minimal Matrigel + defined growth factors	Luminal/basal layers; growth factor-dependent expansion; ductal branching	Lineage fate transitions, growth factor modulation, tissue engineering	2022	Sahu et al. (2022)

#### 2.3.1 Insights from mouse ESC-derived organoids

Parallel research using mouse PSCs has provided complementary insights. Sahu et al. used spatiotemporal regulation of growth factors to direct the differentiation of mouse ESCs into multi-lineage ESC-derived mammary organoids (MEMOs). These organoids were capable of secreting milk and contained various mammary-specific microenvironmental cells, offering new insights for extending this strategy to human-derived PSCs in future studies (Sahu et al., 2022).

#### 2.3.2 Establishment and maturation of human iPSC-derived models

For human iPSCs, a two-step method generates mammary-like organoids expressing luminal and basal markers and responsive to prolactin (Qu et al., 2017). Co-culture with human foreskin fibroblasts enhances branching and duct formation, and xenotransplantation improves architectural maturity (Dai et al., 2022).

PSC-derived mammary organoids provide a renewable and experimentally controllable platform for modeling early mammary development, as they enable stepwise differentiation from pluripotent cells into mammary epithelial lineages. Human iPSC-derived mammary-like organoids express both luminal and basal markers and produce milk proteins in response to prolactin, demonstrating partial lineage fidelity and functional maturation. Their maturity can be further enhanced by fibroblast co-culture, which promotes branching and multilayered duct-like organization, and by xenotransplantation, which improves architectural maturation. Nevertheless, compared with primary human mammary tissue and adult tissue-derived organoids, PSC-derived models often retain relatively immature transcriptional profiles, simplified tissue architecture, and insufficient stromal components, including endothelial cells, fibroblasts, adipocytes, and immune cells. Future studies should systematically benchmark their cellular composition, lineage-specific transcriptional profiles, hormone receptor expression, hormonal responsiveness, and lactational function against primary human mammary tissue.

## 3 Construction strategies for BCOs

**Fig. 3:**
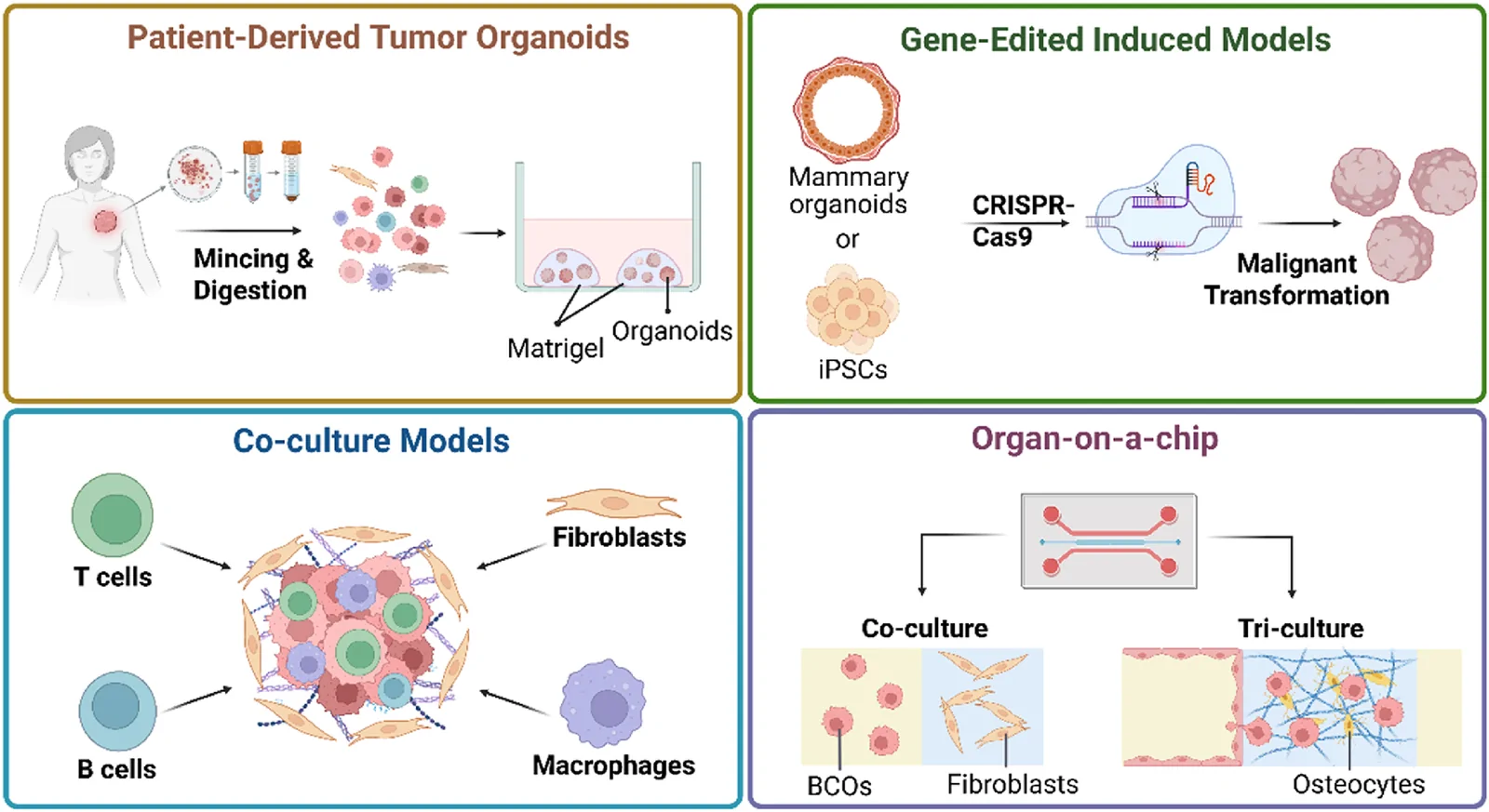
**Principal strategies for constructing BCOs. Upper left:** Patient-derived tumor organoids are generated by mechanical mincing and enzymatic digestion of breast tumor tissues, followed by embedding of isolated tumor-cell clusters in Matrigel or another basement membrane extract. **Upper right:** Gene-editing-induced models are established by introducing defined genetic alterations into normal mammary organoids or induced pluripotent stem cell-derived mammary cells, commonly using CRISPR–Cas9, to model malignant transformation. **Lower left:** Co-culture models incorporate immune and stromal components, including T cells, B cells, fibroblasts, and macrophages, to reconstruct selected tumor-microenvironment interactions. **Lower right:** Organoid-on-a-chip platforms provide controlled microfluidic conditions for co-culture or tri-culture of breast cancer organoids with fibroblasts, vascular or bone-associated cells, and other microenvironmental components. **BCOs**, breast cancer organoids; **CRISPR–Cas9**, clustered regularly interspaced short palindromic repeats-associated protein 9; **iPSCs**, induced pluripotent stem cells.

Over the past decade, organoid technology has advanced from the proof-of-concept stage to a routine tool in preclinical and translational research. In breast cancer, four complementary strategies have emerged: patient-derived tumor organoids (PDOs) from fresh tumor tissue, gene-editing-induced models, microenvironment-reconstructed platforms that incorporate immune and stromal components, and organ-on-a-chip technology (Fig. 3). Each offers distinct advantages suited to different research purposes and applications.

### 3.1 Simple tumor organoid models

The most widely established breast cancer organoid models are those consisting primarily of tumor epithelial cells, obtained either directly from patient tissues or generated through genetic manipulation of normal mammary organoids or pluripotent stem cells. Both approaches yield scalable, reproducible models suitable for high-throughput drug screening and mechanistic studies, albeit with different strengths and limitations.

#### 3.1.1 Patient-derived tumor organoids (PDOs)

PDOs represent one of the most widely used breast cancer organoid models. Breast cancer tissues from surgical resection or needle biopsy are mechanically minced and enzymatically digested (e.g., with collagenase) to isolate epithelial cell clusters, which are subsequently embedded into basement membrane extract (e.g., Matrigel) to form three-dimensional dome structures and cultured in medium supplemented with specific growth factors (Table 4).

**Table 4:** BCOs derived from patient samples.

Species	Source of cells	Construction method	Key features	Application	Year	Ref.
Human	Patient's primary or metastatic tumor cells	3D culture in basement membrane extract (e.g., Matrigel, collagen)	Breast cancer tissues obtained from surgical resection or needle biopsy are mechanically minced and enzymatically digested (e.g., with collagenase) to isolate epithelial cell clusters. These clusters are subsequently embedded into basement membrane extract (e.g., Matrigel) to form three-dimensional dome structures and cultured in medium supplemented with specific growth factors. PDOs possess high fidelity.	Drug resistance mechanisms and drug treatment testing	2020	Whittle et al. (2020)
				Personalized drug guidance	2021	Chen et al. (2021)
				Drug screening	2022	Sun et al. (2022)
				Drug testing	2023	Madorsky Rowdo et al. (2023)
				Drug screening	2023	Zhang et al. (2023)
				Tumorigenesis studying and potential carcinogenic factor screening	2024	Kong et al. (2024)
				Drug screening	2024	Lin et al. (2024)
				Drug screening	2024	Soosainathan et al. (2024)
				Living Biobank	2024	Wu et al. (2024)
				Living Biobank	2025	Kim et al. (2025)
				Living Biobank	2025	Madorsky Rowdo et al. (2023)
				Tumor metastasis mechanisms	2025	Ou-Yang et al. (2025)
				Drug resistance and potential targets screening	2025	Tessier et al. (2025)
	Patient's circulating tumor cells		CTCs were sorted from blood samples	Tumor metastasis mechanisms	2025	Würth et al. (2025)

Since Lee et al. first reported a three-dimensional culture protocol for normal and malignant mammary cells in 2007, culture conditions have been continuously optimized (Lee et al., 2007). Key components for BCO culture media currently include B27 supplement, N-acetylcysteine, nicotinamide, EGF, R-spondin-1, Noggin, Neuregulin-1, Y-27632, and A83-01. B27 is a serum-free supplement containing vitamins and antioxidants. N-acetylcysteine reduces oxidative stress and supports cell survival, whereas nicotinamide, an active form of vitamin B3, participates in cellular energy metabolism. Y-27632 inhibits Rho-associated protein kinase and reduces dissociation-induced apoptosis (Watanabe et al., 2007), while A83-01 inhibits transforming growth factor-β signaling and supports stem-cell self-renewal (Tojo et al., 2005). Together, these components maintain BCO viability and propagation.

In 2018, Sachs et al. created a living biobank consisting of over 100 breast cancer organoid lines, which effectively recapitulate the diversity of breast cancer (Sachs et al., 2018). In 2021, Dekkers et al. further developed a standardized and reproducible experimental workflow, facilitating the construction of organoid models from human normal or breast cancer tissues, which also supports long-term culture, gene editing, and xenotransplantation (Dekkers et al., 2021). Establishment success rates of PDOs exceed 80% across molecular subtypes (ER^+^, HER2^+^, TNBC) (Fitzpatrick et al., 2023; Ryu et al., 2025; Würth et al., 2025), with organoids closely retaining key morphological, histological, and receptor expression features (ER, PR, HER2) of primary tumors, making them ideal models for personalized therapeutic studies.

Despite the widespread application of PDOs, several critical challenges remain to be addressed. A major concern is the establishment of standardized culture procedure and conditions that are broadly applicable across patients and molecular subtypes. Although BCOs can preserve the fidelity of HR^+^ and HER2^+^ tumors, oxygen-rich culture conditions may deplete stem-like hypoxic subpopulations in TNBC, thereby underestimating drug resistance and overestimating drug sensitivity (Jia et al., 2024). Long-term genetic and phenotypic stability, including morphology, histology, and receptor expression, also remains a concern during extended expansion. For example, prolonged culture of luminal BCOs in some commercial media can result in loss of ER, PR, and HER2 expression (Zhang et al., 2026), while establishment efficiency remains low (Przanowska et al., 2024). Standardized long-term culture protocols are therefore required to maintain PDO fidelity.

#### 3.1.2 Gene editing-induced breast cancer organoid models

Gene-edited models, using CRISPR-Cas9 to introduce or delete specific genes in normal mammary organoids or iPSCs, have emerged as powerful tools for recapitulating key oncogenic processes including tumor initiation, progression, therapy resistance, and metastasis (Table 5).

**Table 5:** Gene-editing-induced BCOs models.

Species	Source of cells	Construction method	Key features	Application	Year	Ref.
Human	Human induced pluripotent stem cells	Sequentially knock out four frequently mutated tumor suppressor genes in normal human epithelial mammary organoids	Simulate the progression of tumorigenesis	Tumorigenesis studying	2019	Dekkers et al. (2019)
Mouse	Normal mammary epithelial cells	Specifically knock out the *Trp53* and *Brca1* genes in the Krt8^+^ luminal cells			2019	Wang et al. (2019)
Human	Human induced pluripotent stem cells	Differentiate BC-hiPSCs reprogrammed from cancer cells of breast cancer patients into mammary epithelial cells and form mammary-like organoids		Tumor heterogeneity and drug testing	2025	Weddle et al. (2025)

One common strategy involves direct gene editing in normal mammary organoids. In 2019, Wang et al. knocked out *Brca1* in mouse luminal mammary organoids and successfully observed a “luminal-to-basal/mesenchymal” phenotypic transition, leading to the construction of BCOs (Wang et al., 2019). In 2019, Dekkers et al. sequentially knocked out four frequently mutated tumor suppressor genes (*TP53*, *PTEN*, *RB1*, and *NF1*) in normal human epithelial mammary organoids, achieving the stepwise evolution from normal mammary cells to ER ^+^ breast cancer within an *in vitro* system (Dekkers et al., 2019).

More recently, Weddle et al. designed a novel method to generate BC-hiPSCs reprogrammed from cancer cells of breast cancer patients with various gene variants, and then differentiated them into mammary epithelial cells and formed mammary-like organoids (Weddle et al., 2025). They demonstrated that organoids with distinct gene variants possess different sensitivities to PARP inhibitors.

Gene-editing organoids are a powerful tool in mimicking tumorigenesis, but they are limited by technical complexity, low delivery efficiency, and batch variability.

### 3.2 Co-culture-derived microenvironmentalized BCOs

The tumor microenvironment (TME), including cancer-associated fibroblasts (CAFs), tumor-associated macrophages (TAMs), various lymphocytes, endothelial cells, pericytes, extracellular matrix (ECM), and a variety of cytokines and chemokines, critically influences tumor behavior and therapy response. A wealth of clinical and experimental evidence indicates that the immune cell infiltration and CAFs/ECM-mediated mechanical signals influence the molecular subtypes, metastasis, therapeutic resistance, and immune evasion (Junttila & de Sauvage, 2013; Klemm & Joyce, 2015; Zhang & Zhang, 2020). Therefore, precisely recreating tumor-microenvironment interactions *in vitro* is of paramount importance for understanding drug resistance mechanisms and optimizing immunotherapy strategies. Two primary technical approaches exist: exogenous reconstruction of immune components and endogenous preservation of native immune components, with the former being more commonly used in breast cancer research (Table 6).

**Table 6:** Microenvironment-reconstituted BCO models.

Species	Source of cells	Construction method	Key features	Application	Year	Ref.
Human	MCF7 and MDA-MB-231 tumor cells	Fibroblasts and BCOs co-culture, non-contact, with an epithelial cell to fibroblast ratio of 1:3	Organoids co-cultured with fibroblasts	Tumor progression mechanisms	2020	Cheng et al. (2020)
Human	Patient's primary tumor cells	Samples were gentle pipetted and sliced into small fragments which were embedded in basement membrane matrix hydrogel to form 3D structure	PDOs retaining tumor microenvironment	Personalized drug testing	2021	Voabil et al. (2021)
Mouse	E0771 tumor cells	Organoids were collected and mixed with corresponding CD8^+^ T cells at a ratio of 1:200 in the ultra-low attachment microplates	Organoids co-cultured with T cells	Drug testing	2021	Zhou et al. (2021)
Human	MDA-MB-468 tumor cells					
Human	Patient's primary tumor cells					
Human	Patient's primary tumor cells	PBMCs were mixed with single-cell dissociated organoids at a 20:1 ratio	PDOs co-cultured with PBMC	Drug testing	2022	Huang et al. (2022)
Mouse	KB1P tumor cells	T cells were mixed with tumor cells at a 1:3 ratio	Organoids co-cultured with T cells	Drug testing and mechanism exploration	2023	Vennin et al. (2023)
Human	Patient's primary tumor cells	T cells were mixed with tumor cells at a 2:1 ratio and embedded into basement membrane extract				
Human	MCF-7 cells	M2 macrophages and MCF-7 cells were mixed and co-cultured in cryogel at a 2:1 ratio	Organoids co-cultured with TAMs	Tumor progression mechanisms	2024	Xu et al. (2024)
Human	MDA-MB-231, MDA-MB-468, and T-47D cells	Breast cancer cells and fibroblasts were mixed at a 1:1 ratio and were performed using 3D-on-top methods	Organoids co-cultured with fibroblasts	Tumor progression mechanisms and therapy resistance mechanisms	2025	Klosowski et al. (2025)
Human	Patient's primary tumor cells	Samples were gently pipetted and sliced into small fragments, which were embedded in basement membrane matrix hydrogel to form a 3D structure	PDOs retaining tumor microenvironment	Tumor heterogeneity	2025	Kumari et al. (2025)
Human	Patient's primary tumor cells	Adipocytes were placed in a tissue Transwell culture plate of tumor organoids at a 1:1 ratio of adipocyte media and breast cancer organoid media	PDOs co-cultured with adipocytes	Tumor progression mechanisms	2025	Nguyen et al. (2025)
Human	Patient's primary tumor cells	Organoids single cells mixed with CAFs at a 1:1 ratio were co-cultured in low-attachment plates to form spheroids, and resuspended in matrix gel	Organoids co-cultured with fibroblasts	Tumor progression mechanisms and drug testing	2025	Shi et al. (2025)

In 2020, Cattaneo et al. established a foundational tumor organoid–T cell co-culture system (Cattaneo et al., 2020). Building on this, in 2021, Zhou et al. constructed a standardized and reproducible BCOs–T cell co-culture platform for high-throughput screening of epigenetic drugs that enhance anti-tumor immunity (Zhou et al., 2021). Later, Vennin et al. developed a co-culture platform combining BCOs and peripheral blood mononuclear cell (PBMC)-derived T cells to investigate the mechanism of action of taxanes in 2023 (Vennin et al., 2023).

Given the central role of CAFs, Cheng et al. co-cultured CAFs with BCOs by culturing fibroblasts in an insert above the organoids at a ratio of 3:1 to explore CAF-mediated tumor progression (Cheng et al., 2020). Following this, in 2025, Shi et al. established a CAF–PDO co-culture model by mixing CAFs with single tumor cells at a 1:1 ratio and culturing under low-adhesion conditions to form mixed organoids which recapitulated ECM architecture and mechanical sensing (Shi et al., 2025). Additional studies have further explored lung-metastatic breast cancer by co-culturing BCOs with lung fibroblasts (Klosowski et al., 2025).

Moreover, in 2024, Xu et al. successfully achieved co-culture of BCOs and TAMs using alginate cryogel as a biomimetic ECM scaffold (Xu et al., 2024). In 2025, Nguyen et al. conducted research on the relationship between obesity, metabolism, and breast cancer by establishing a co-culture system of BCOs and engineered adipocytes (Nguyen et al., 2025).

Furthermore, a recent study in 2025 adopted a purely mechanical dissociation approach to construct 3D organoid models, avoiding enzymatic disruption of cell–cell interactions and ECM integrity (Kumari et al., 2025). This approach preserved diverse cellular components, including B cells (CD20^+^), leukocytes, mesenchymal stem cells (CD73^+^, CD90^+^, CD105^+^), vascular endothelial cells (CD34^+^, CD105^+^), and fibroblasts, faithfully recapitulating the histology and intratumoral heterogeneity of the primary tumor.

The evolution of BCO co-culture systems from simple binary models to complex multi-component platforms represents a critical advance in simulating the TME. These models have demonstrated tremendous potential for studying tumor-stroma interactions, driver gene functions, and preclinical evaluations of immunotherapy. However, each co-culture strategy possesses different advantages and limitations, and fits in different scenarios. The exogenous reconstruction approach offers high experimental controllability, enabling researchers to dissect the individual roles of specific cell types, such as CAFs or TAMs, in tumor progression and therapy resistance. But it lacks the complexity of real TME *in vivo*, which includes diverse immune cell subsets, vascular networks, and ECMs. Conversely, endogenous preservation is superior in preserving the original histological architecture and intercellular interactions, thereby providing a more favorable platform for studying the complex interplay between cancer cells and their native stroma and for predicting immunotherapeutic responses. However, the long-term cultivation of such organoids remains a bottleneck.

Looking forward, emerging vascularized organoid models and multicellular culture systems provide more possibilities. For instance, the integration of microvascular networks enables the study of tumor hematogenous metastasis (Leonteva et al., 2026) and the assessment of drug responses (Lo Cicero et al., 2026), while the inclusion of diverse immune populations and other ECMs provides a platform to investigate dynamic immune surveillance, the mechanisms of immune evasion, and immunotherapies. Collectively, BCO co-culture models are evolving from single-cell-type to multicellular systems, and are increasingly incorporating diverse advanced technologies to build multi-component TME models that closely simulate the real conditions within the body.

### 3.3 Microfluidic dynamic culture platforms (organoid-on-a-chip)

Organoid-on-a-chip (OoC) platforms integrate microfluidics to overcome limitations of static culture conditions, enabling controlled perfusion, shear stress, oxygen and nutrient gradients, and multicellular co-culture within sub-millimeter chip chambers (Park et al., 2019; Ruzycka et al., 2019).

Typical OoC configurations include dual-channel “vascular–tissue” polydimethylsiloxane (PDMS) chips or 96-well array chambers pre-coated with Matrigel or patterned hydrogels to maintain organoid polarity and control diameter variation. Shear stress can be precisely regulated within 0.1-10 dyn/cm^2^, and perfusable microvascular networks can be established within 3-7 days. The fabrication workflow generally involves designing microchannel structures (e.g., 100 μm width, 25 μm height) using AutoCAD, creating SU-8 photoresist masters through photolithography, casting PDMS molds and bonding them to glass slides for sealing, followed by injection of cell-matrix suspensions and connection to microfluidic pumps. Organoids are then dynamically cultured for 7-14 days at 37 °C, enabling in situ drug administration and real-time imaging.

Researchers have designed diverse chip models depending on the research questions. For example, Truong et al. utilized a microfluidic platform to construct a “breast cancer tumor cell-fibroblast” model revealing that intercellular interactions significantly enhance cancer cell invasion in a GPNMB-dependent manner (Truong et al., 2019). In 2025, Ravi et al. developed a tri-culture 3D breast TME-on-a-chip (TMEC) model to evaluate the progression of triple-negative breast cancer cells in the presence of patient-derived CAFs and macrophages (Ravi et al., 2025). Furthermore, emerging multi-organ chip systems allow co-culture of breast cancer PDOs with immune cells and endothelial cells, enabling the simultaneous simulation of the tumor-immune-vascular ternary interaction. For instance, in 2014, Bersini et al. constructed the first “human bone-vascular endothelial-breast cancer” triadic 3D model on a microfluidic chip, enabling real-time visualization of immune cell extravasation and identifying the CXCL5/CXCR2 axis as a potential therapeutic target (Bersini et al., 2014) (Table 7).

**Table 7:** Organ-on-a-chip models.

Species	Source of cells	Construction method	Key features	Application	Year	Ref.
Human	Patient's primary tumor cells	Human bone-vascular endothelial-breast cancer triadic 3D model on a microfluidic chip	Organoids with bone and endothelial cells	Potential targets screening	2014	Bersini et al. (2014)
	SUM-159 breast cancer tumor	Tumor cells were encapsulated in type I collagen, polymerized at 37 °C for 8 min in a humidified incubator	Organoids with CAFs	Tumor progression mechanisms	2019	Truong et al. (2019)
	Patient's primary tumor cells	Tumor-on-chip platform in which breast organoids are placed and cultured on an adjacent endothelialized microchannel with constant CAR-T cell perfusion	Organoids with CAR-T	Drug testing	2024	Maulana et al. (2024)
	Patient's primary tumor cells	Human breast cancer-fibroblasts-immune cells triadic 3D model on a microfluidic chip	Organoids with CAFs and macrophages	Tumor progression mechanisms	2025	Ravi et al. (2025)

In summary, OoC technology introduces new dimensions and possibilities for co-culture models, enhancing structural complexity and functional maturity of organoids while providing a physiologically relevant tool for studies of organogenesis, advancing drug development, and modeling human diseases. Despite its considerable potential, challenges such as high costs and technical complexity hinder its large-scale application.

### 3.4 Comparison of three breast cancer organoid platforms

In summary, the three classes of breast cancer organoid models present distinct strengths and limitations. Simple PDO models retain substantial tumor heterogeneity, and the construction process is fast and low-cost, making them suitable for high-throughput drug screening. However, the model completely lacks the native microenvironment, limiting their fidelity and applications. Co-culture systems, by integrating single or multiple TME components, partially reconstitute intercellular crosstalk, which makes them amenable to immune-related research, such as the evaluation of immunotherapy. Compared to simple PDO models, co-culture systems are more biomimetic, but the technology is more complex. Microfluidic OoC models integrate dynamic perfusion, multi-cellular or multi-organ architectures, and, in some designs, vascularized networks, thereby achieving higher physiological fidelity. These elaborate systems are appropriate for real-time monitoring of drug delivery or tumor evasion and metabolism. Nevertheless, the high costs and complex technical requirements hinder their high-throughput and large-scale application. Therefore, the selection of the most appropriate model should be guided by the specific research context, carefully balancing physiological relevance against practical feasibility. Future efforts should be directed towards developing standardized, clinically translatable platforms that synergistically combine the advantages of these approaches while addressing their respective bottlenecks.

## 4 Applications of mammary organoids

**Fig. 4:**
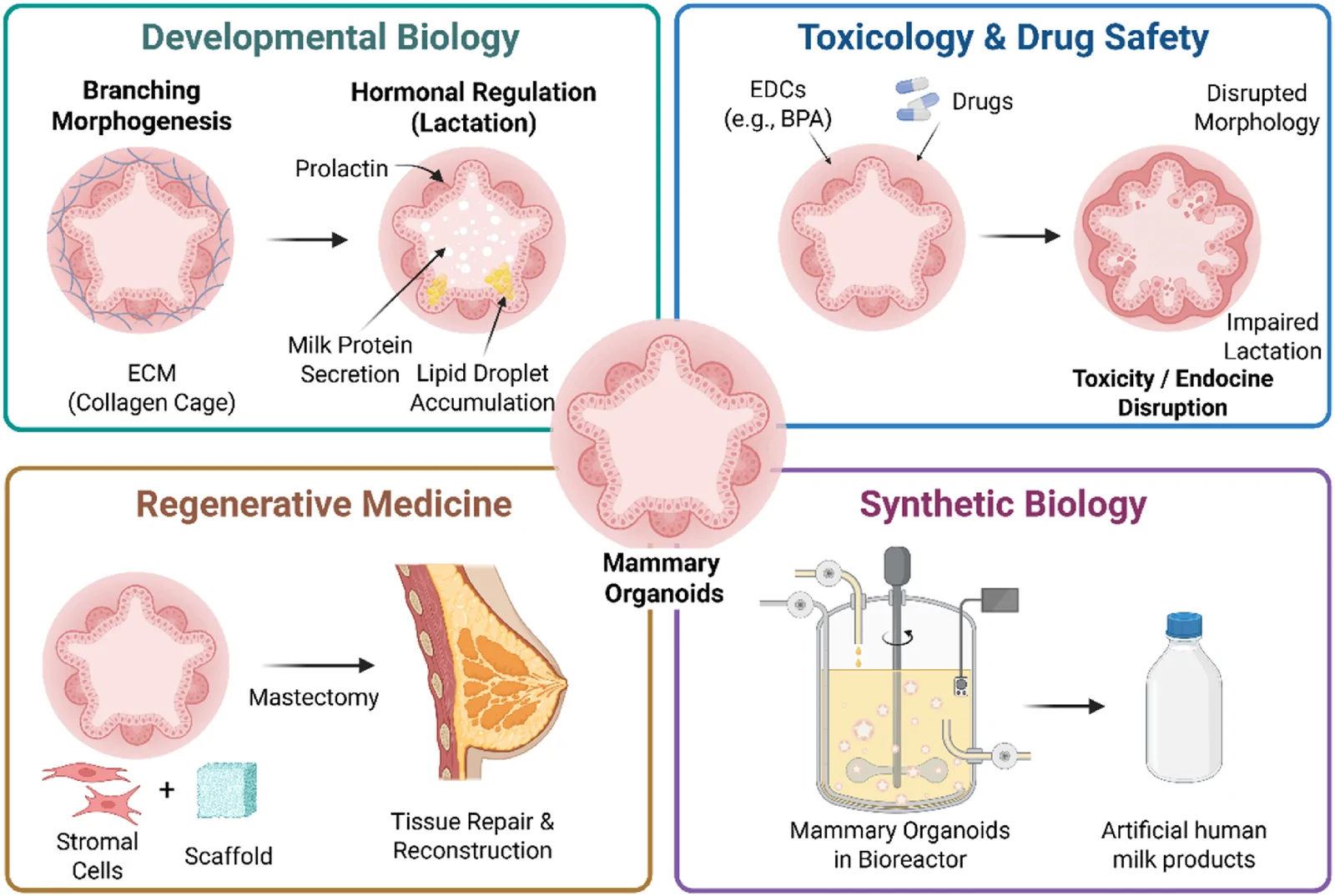
**Major applications of mammary organoids.** Mammary organoids provide versatile experimental platforms for developmental, toxicological, regenerative, and synthetic-biology studies. **Upper left:** In developmental biology, mammary organoids reproduce extracellular matrix-dependent branching morphogenesis and hormone-regulated lactogenic differentiation, including prolactin-induced milk-protein secretion and lipid-droplet accumulation. **Upper right:** In toxicology and drug-safety studies, organoids are exposed to endocrine-disrupting chemicals or candidate drugs to evaluate alterations in tissue morphology, epithelial organization, and lactational function. **Lower left:** In regenerative medicine, mammary organoids can be combined with stromal cells and biomaterial scaffolds to investigate mammary-tissue repair and reconstruction after surgical injury or mastectomy. **Lower right:** In synthetic biology, mammary organoids can be expanded and functionally maintained in bioreactors for the production of milk-associated components or artificial breast-milk products. **BPA**, bisphenol A; **ECM**, extracellular matrix; **EDCs**, endocrine-disrupting chemicals.

Mammary organoids have been applied in various fields, including developmental biology, drug safety evaluation, toxicology, synthetic biology, and regenerative medicine (Fig. 4).

### 4.1 Applications in developmental biology research

Mammary organoids reconstruct mammary gland architecture *in vitro* while retaining multilineage characteristics, providing a tractable platform to study development.

#### 4.1.1 Branching morphogenesis

Mammary organoids enable dissection of branching morphogenesis. Early studies established that ECM composition and growth factor signaling determine branching behavior (Vidi et al., 2013). Using human mammary organoids with live imaging and biomechanical analysis, Buchmann et al. discovered that branch tip cells undergo oscillatory collective migration, reorienting collagen fibers to form a “collagen cage” that directs branch elongation (Buchmann et al., 2021). Subsequent standardized organoid models have systematically recapitulated distinct branching stages (Caruso et al., 2022), and cellular dynamics studies showed that branch tip cells drive ECM remodeling through E-cadherin–dependent collective migration (Hutterer et al., 2022).

#### 4.1.2 Hormonal regulation

Mammary gland development is highly dependent on hormonal regulation, with estrogen, progesterone, and prolactin playing pivotal roles at distinct physiological stages. Beyond their role in branching morphogenesis, mammary organoids have been widely employed in studying hormonal regulation in mammary development and disease because of their preserved sensitivity to hormonal cues.

In mouse organoids, prolactin stimulation upregulates milk proteins and induces lactation-associated features, while withdrawal triggers involution-like changes with upregulation of matrix remodeling genes (Sumbal et al., 2020). Mouse ESC-derived organoids similarly produce milk proteins in response to prolactin (Sahu et al., 2022). The system also allowed parsing of prolactin (differentiation) and oxytocin (contraction) axes (Sumbal et al., 2020). Yuan et al. developed a murine mammary mini-gland system that recapitulates puberty, estrous cycle, and pregnancy/lactation, with long-term stability dependent on dynamic hormonal cycling (Yuan et al., 2023). Single-cell transcriptomics confirmed that combined estrogen, progesterone, and prolactin expand specific luminal progenitor populations, faithfully capturing *in vivo* lineage-specific regulation (Ortiz et al., 2023).

#### 4.1.3 Lineage fate transitions

As the application of mammary organoids in studying branching morphogenesis and hormonal responsiveness has matured, research has progressively shifted from focusing on “morphological outputs” to exploring “lineage specification and fate plasticity” at the cellular level. Organoid systems combined with lineage tracing have illuminated Notch signaling in mammary lineage specification. Notch activation expands luminal progenitors and promotes luminal differentiation, while inhibition increases stem cell numbers (Bouras et al., 2008). Notch activation in basal cells induces basal-to-luminal conversion (Merle et al., 2025). Using an *in vitro* dynamic mammary mini-gland system with lineage tracing, Yuan et al. captured the developmental transition from embryonic bipotency to postnatal unipotency, providing a controllable platform to dissect fate specification timing and mechanisms (Yuan et al., 2023).

Collectively, these studies demonstrate that mammary organoids can faithfully recapitulate lineage transitions observed during normal mammary development and, importantly, provide a controllable platform to dissect the signaling pathways and temporal dynamics that govern lineage specification and fate plasticity at the cellular level.

### 4.2 Applications in drug safety evaluation

In recent years, organoid technology has demonstrated significant potential in the field of drug toxicity assessment, although applications of mammary organoids remain in their infancy. Existing studies on tissues such as the liver, kidney, heart, and brain have shown that organoid systems can more accurately recapitulate human tissue architecture and function, thereby enhancing the predictive validity of drug safety testing (Yang et al., 2024). Furthermore, research has suggested that combined screening using organoids derived from both normal tissue and tumors can identify tumor-selective responses as well as potential toxicities to normal tissues, offering a valuable reference for detecting drug-specific side effects on normal mammary epithelium (Calandrini & Drost, 2022).

In addition, Sumbal et al. developed a mammary organoid system from mouse tissue that, under stimulation with FGF2 and prolactin, was able to simulate both lactation and regression programs (Sumbal et al., 2020). This finding indicates the potential application of this platform for evaluating the risk of drug-induced disruptions of lactation function (e.g., by certain antipsychotic medications). Although no published studies have yet provided a comprehensive assessment of the systemic effects of drugs and their metabolites on mammary function and healthy mammary architecture, the integration of these technologies and models suggests that mammary organoids hold great promise as a platform for evaluating breast-specific drug toxicity and lactation-associated side effects.

### 4.3 Applications in toxicology research

The effects of endocrine-disrupting chemicals (EDCs) on mammary tissue, particularly their roles in perturbing mammary development and promoting breast cancer, have received increasing attention in recent years. Mammary organoids provide a tractable platform for evaluating the toxicity of EDCs on mammary tissue. Exposure to bisphenol A or bisphenol S induced dose-dependent morphological alterations in mammary alveoli associated with carcinogenesis (Winkler et al., 2022). Similar organoid-based approaches have been applied to study other EDCs, though accurately modeling chronic low-dose exposure remains challenging (Hwang et al., 2025).

Further studies are needed to evaluate how these environmental pollutants affect mammary stem cells over time and how they may contribute to breast development and tumorigenesis through genetic mutations or transcriptional reprogramming.

### 4.4 Applications in synthetic biology

With the advancement of organoid technology, mammary organoids have reached increasing levels of functional maturation. The primary physiological role of the mammary gland is milk secretion to nourish offspring. However, a substantial proportion of infants worldwide cannot be exclusively breastfed. While existing formula milk provides basic nutrition, it fails to replicate the complexity and bioactivity of human breast milk. Against this backdrop, the emergence of artificial breast milk may disrupt the conventional formula industry and offer new options for families unable to breastfeed.

Artificial breast milk is an emerging field that aims to simulate and reproduce the nutritional components and bioactive properties of human milk through biotechnological approaches, thereby meeting the needs of newborns who cannot access maternal breastfeeding. Currently, several companies in the infant formula industry are investing in this direction, primarily using primary mammary epithelial cells or stem cells. For example, Biomilq has developed a system using cells extracted from human breast tissue and milk, cultured in bioreactors designed to mimic the mammary environment, thereby inducing secretion of milk components.

Compared with isolated mammary cells, mammary organoids offer superior advantages in terms of cellular composition, tissue architecture, and physiological function. Consequently, they hold strong potential to drive transformative progress in the artificial breast milk industry and accelerate its development.

### 4.5 Applications in regenerative medicine and tissue repair

Mammary tissue often faces the challenge of reconstruction following breast cancer treatment, particularly after mastectomy. Traditional breast reconstruction methods rely on implanting prostheses or autologous fat grafting, but these approaches do not fully restore the functional structure of the mammary gland. Mammary organoids, by reconstructing mammary tissue architecture *in vitro*, offer new possibilities for breast function restoration and tissue repair.

Repairing mammary tissue requires not only the restoration of its morphological structure but also the recovery of its physiological functions, such as lactation and hormonal responsiveness. In this regard, mammary organoids provide an ideal research platform that can simulate both the structure and function of mammary tissue, while also exploring strategies to promote mammary tissue regeneration after surgery. Mammary organoids co-cultured with fibroblasts or adipocytes show enhanced proliferation and branching, offering potential for functional breast reconstruction after mastectomy (Dai et al., 2022). Dynamic culture systems that simulate pubertal, cyclic, and lactational states provide a foundation for developing tissue-engineered mammary constructs (Yuan et al., 2023).

## 5 Applications of BCOs

**Fig. 5:**
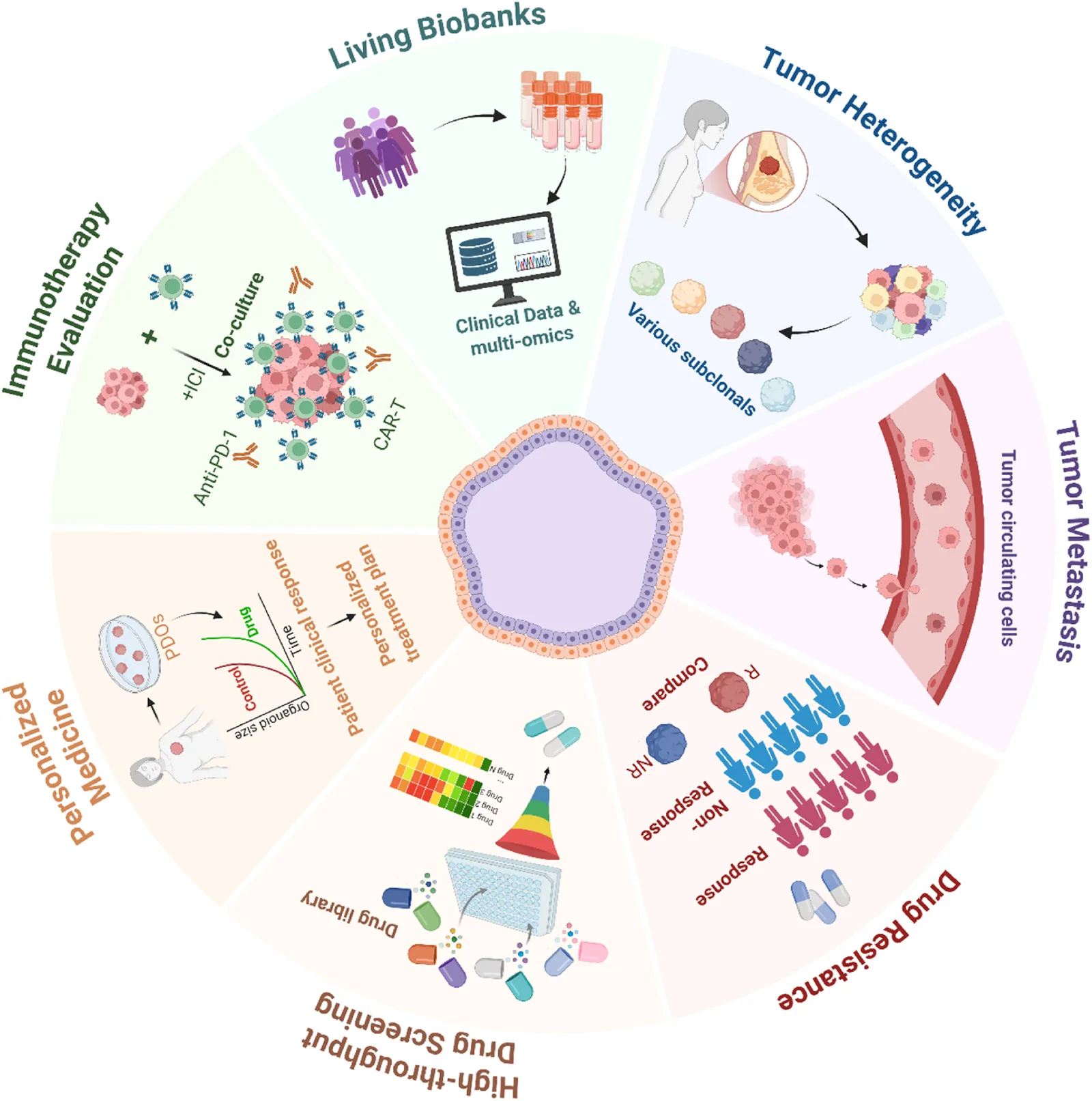
**Major applications of BCOs.** Breast cancer organoids preserve clinically relevant features of primary tumors and support a broad range of basic, preclinical, and translational applications. The sectors, shown clockwise from the top, illustrate the use of breast cancer organoids for establishing living biobanks linked to clinical and multi-omics information; modeling interpatient and intratumoral heterogeneity across breast cancer subclones; investigating tumor-cell dissemination and metastatic progression; studying heterogeneous drug responses and mechanisms of drug resistance; performing high-throughput screening of drug libraries and candidate combinations; conducting patient-specific drug-sensitivity testing to support precision treatment selection; and evaluating immunotherapeutic strategies through co-culture with immune cells, including immune-checkpoint inhibition and chimeric antigen receptor T-cell therapy. **BCOs**, breast cancer organoids; **CAR-T**, chimeric antigen receptor T cells; **ICI**, immune-checkpoint inhibitor; **PDOs**, patient-derived tumor organoids.

Organoids preserve the histological, genomic, and functional characteristics of primary tumors over long-term *in vitro* culture, making them a pivotal tool for preclinical breast cancer research. They provide an unprecedented and precise platform for disease modeling and mechanism investigations, as well as for high-throughput drug screening, personalized drug sensitivity testing, and immune microenvironment studies (Fig. 5).

### 5.1 Living biobanks

The breast cancer organoid banks are repositories that systematically collect, culture, and preserve three-dimensional living tumor models derived from breast cancer patients encompassing various subtypes, stages, and treatment phases, and integrate them with comprehensive clinical, pathological, and molecular information. Such clinical annotations enable researchers to correlate the laboratory performance of organoids with clinical outcomes, thereby validating the reliability of organoids as predictive models.

In 2018, Sachs et al. established “A Living Biobank of BCOs” comprising over 100 organoid lines (Sachs et al., 2018). In 2025, Kim et al. created the first biobank of BCOs sourced from malignant pleural effusion (MPE) (Kim et al., 2025). In 2024, Wu et al. established the first breast cancer PDO biobank in China using samples from a large cohort of patients from northwest China, which is comprised of 60 organoids from 75 breast samples (Wu et al., 2024). In 2025, Madorsky Rowdo et al. established a PDO biobank from African American and African populations containing 77 PDOs from 237 samples (Madorsky Rowdo et al., 2025). All of these studies found that BCOs recapitulated the histopathological features of the primary tumor tissues. This provides researchers with valuable resources for long-term studies without requiring continuous patient involvement.

### 5.2 Disease modeling and mechanism research

As a revolutionary *in vitro* model, BCOs provide a powerful platform for studying the initiation, progression, and heterogeneity of breast cancer. Their greatest advantage lies in their ability to retain the tissue architecture, molecular characteristics, and genetic background of the primary tumor, providing an unparalleled opportunity for mechanism research.

#### 5.2.1 Tumorigenesis mechanisms

The integration of organoid culture with gene-editing technologies, particularly CRISPR-Cas9, offers a powerful platform for functionally validating the multi-step progression of breast cancer and enables real-time tracking of molecular switches driving metastasis and drug resistance.

Researchers can reproduce the tumorigenesis of breast cancer *in vitro* by sequentially knocking out breast cancer-related tumor suppressor genes in normal breast organoids (Dekkers et al., 2019). Gene-edited organoid models can elucidate the dynamic process of tumor initiation and the impact of developmental stages on tumor phenotypes. For instance, in 2023, Yuan et al. constructed an mCherry-LSL-PyMT-GFP expression vector to introduce PyMT oncogene expression into normal mammary organoids, enabling direct observation of tumor initiation dynamics (Yuan et al., 2023). Further, in 2025, Liu et al. developed a breast cancer model using iPSCs derived from patients with germline *BRCA1* mutations, representing the first human-derived iPSC breast cancer model and revealing gene *S100P* as an oncogene downstream of *BRCA1* that drives breast cancer stemness and tumorigenesis (Liu et al., 2025).

Moreover, organoids can be used to screen for potential carcinogenic factors that may lead to tumorigenesis. For example, in 2024, Kong et al. treated PDOs with or without the glycolytic metabolite methylglyoxal (MGO), indicating that MGO can transiently disable the tumor suppressor gene *BRCA2*, which is probably related to cancer evolution (Kong et al., 2024).

In conclusion, gene-edited organoid models provide a unique platform to study specific gene mutations in breast cancer by enabling precise control over the order and combination of mutations introduced. This allows for the elucidation of synergistic interactions during tumorigenesis in normal cells with defined genetic backgrounds, avoiding the interference from accumulated unknown mutations typical of traditional cell lines.

#### 5.2.2 Tumor metastasis and drug resistance mechanisms

In chemotherapy resistance research, significant progress has been made across different breast cancer subtypes. In 2025, Tessier et al. employed TNBC PDOs to reveal the crucial role of EMT-induced primary cilia formation in chemotherapy resistance, proposing the “EMT-cilia-Hedgehog” signaling axis as a promising druggable target (Tessier et al., 2025). Similarly, in 2020, Whittle et al. found that RB1-mutant ER^+^ PDOs were resistant to CDK4/6 inhibitors such as palbociclib, but remained sensitive to BCL-2 inhibitors such as ABT-19, highlighting BCL2 as a therapeutic target for CDK4/6 inhibitor-resistant diseases (Whittle et al., 2020). In 2025, Ou-Yang et al. identified the *KAT6B::ADK* fusion gene as a driver of metastasis and resistance in HR^+^/HER2^−^ breast cancer using PDOs for functional validation and clinical translation, confirming that its knockdown reverses the metabolic reprogramming and tamoxifen resistance driven by itself (Ou-Yang et al., 2025).

For metastatic resistance mechanisms, circulating tumor cell (CTC)-derived organoids (CDOs) have emerged as a new tool. In 2025, Würth et al. established the first long-term CDO platform from peripheral blood and pleural or ascitic fluid of metastatic breast cancer patients (Würth et al., 2025). Through CRISPR screening, they identified a “plastic complementary” mechanism between the NRG1-HER3 and FGFR1 signaling axes that drives adaptive resistance of CTCs during the metastatic process, providing a basis for dual-target combination strategies to intervene in metastasis.

#### 5.2.3 Tumor heterogeneity studies

Breast cancer heterogeneity, both intra- and inter-tumor, drives treatment resistance and evolution. Organoid models preserve the clonal diversity of primary tumors, making them key platforms for studying heterogeneity.

Sachs et al. first showed that BCOs retain *TP53* mutation profiles and subclonal structure consistent with the primary tumors after over 6 months of *in vitro* culture (Sachs et al., 2018). Subsequent studies extended this to multiple molecular subtypes, systematically validating their reliability in preserving transcriptomic, epigenomic, and microenvironmental features (Chen et al., 2021; Fang et al., 2024; Fitzpatrick et al., 2023; Weddle et al., 2025; Würth et al., 2025). In 2025, Kim et al. established an MPE-derived breast cancer organoid biobank, integrating whole exome sequencing and single-cell transcriptomics (Kim et al., 2025). They longitudinally tracked clonal evolution across time points, offering an expandable preclinical tool for personalized therapy and resistance prediction.

With the aid of single-cell multi-omics and deep sequencing technologies, researchers can reconstruct subclonal architectures at the organoid level, revealing significant differences in driving mutations, copy number variations, and expression profiles that align with primary tumors. Through timed sampling and computational modeling, organoids recapitulate the entire “mutation-selection-expansion” process under natural or therapeutic conditions, providing a dynamic and reproducible experimental platform to study dominant clone formation and potential therapeutic targets.

### 5.3 Drug screening and personalized drug sensitivity testing

BCOs, replicating primary tumor heterogeneity, also serve as an *in vitro* platform for high-throughput drug screening and personalized sensitivity testing, accurately predicting patient responses to therapies.

#### 5.3.1 High-throughput drug screening

BCOs preserve the histological features, mutation profiles, and subclonal structures of primary tumors, serving as a physiologically relevant platform for high-throughput drug discovery. Sachs et al. developed a standardized culture and phenotypic readout protocol in 384-well plates, enabling high-throughput screening of single or combination therapies (Sachs et al., 2018). On this basis, in 2022, Sun et al. screened 120000 small molecules and identified compound S6, which significantly suppressed SOST expression in organoids in a dose- and time-dependent manner and inhibited tumor bone metastasis, highlighting SOST as a promising target (Sun et al., 2022). Furthermore, in 2023, Zhang et al. screened over 2400 natural products in TNBC PDOs and identified YH677, which blocks the TGF-β/Smad signaling axis to inhibit cancer stem cell self-renewal and reduce tumorigenicity in TNBC-PDX models (Zhang et al., 2023). Similarly, Soosainathan et al. used ER^+^ PDOs to screen 71 compounds and found that the CDK9 inhibitor, AZD4573, synergized with palbociclib to drive tumor regression in endocrine-resistant ER^+^ breast cancer, implicating that the PI3K-AKT-mTOR signaling pathway played a significant role in drug resistance (Soosainathan et al., 2024).

#### 5.3.2 Personalized drug sensitivity testing

PDOs serve as an “*in vitro* surrogate” for real-time drug sensitivity testing, representing one of the most clinically translatable directions for organoid technology. Vlachogiannis et al. established a “PDO-drug-clinical response” triadic validation framework encompassing specimen collection, organoid construction, drug exposure, phenotypic readout, and clinical outcome tracking, which has become a methodological standard across cancer types (Vlachogiannis et al., 2018). Subsequently, in 2021, Chen et al. generated 99 PDOs covering TNBC, HER2^+^, and Luminal A/B subtypes and reported high concordance between PDO drug sensitivity results and prior patient treatment responses (AUC = 80.1%) (Chen et al., 2021).

Numerous studies in breast cancer have confirmed that organoid-based drug sensitivity testing accurately predicts patient responses to chemotherapy, targeted therapies, endocrine treatments, and neoadjuvant therapies (Cui et al., 2024; Madorsky Rowdo et al., 2023; Ye et al., 2024). For instance, Ryu's team established a PDO-PDX-PDXO triadic system and demonstrated that TNBC patients with high WEE1 expression benefit from the WEE1 inhibitor AZD1775, with PDXO serving as a fast and cost-effective tool for personalized drug screening (Ryu et al., 2025). Madorsky Rowdo et al. used PDOs to identify mutant p53 as a key predictive factor for response to PARP inhibitors combined with temozolomide, and also employed organoids for mechanistic analysis and personalized drug screening (Madorsky Rowdo et al., 2023). In 2024, Lin et al. performed drug sensitivity tests on 46 metastatic BCOs and found that the accuracy of predicted clinical response reached 78.4% (Lin et al., 2024). Similarly, Wu et al. compared prior treatment responses of three breast cancer patients with corresponding organoid drug sensitivity results through immunohistochemical analysis and whole-exome sequencing, finding that the organoid responses to drugs were consistent with clinical observations (Wu et al., 2024).

#### 5.3.3 Immunotherapy response prediction

Immunotherapies, such as immune checkpoint inhibitors (ICIs), have shown limited efficacy in breast cancer, with objective response rates of only 5%-10%. A lack of functional models hinders the accurate selection of patients who are likely to benefit. Breast cancer organoid-immune co-culture models can faithfully simulate tumor-immune interactions, providing a powerful platform for immunotherapy response prediction, mechanism research, and personalized treatment strategies.

In 2018, Jenkin et al. developed a 3D microfluidic chip using patient- and murine-derived organotypic tumor spheroids (PDOTS/MDOTS), demonstrating that TBK1/IKKε inhibitors combined with PD-1 blockade significantly enhanced IFN-γ^+^ CD8^+^ T cell infiltration and prolonged mouse survival, enabling assessment of monotherapy and combination strategies (Jenkins et al., 2018). Further, in 2022, Huang et al. utilized PDOs and an autologous PBMC co-culture model to reconstruct tumor-immune interactions and found that DC vaccine HELA-Exos promoted cDC1 infiltration and enhanced CD8^+^ T cell cytotoxicity, significantly inhibiting organoid growth (Huang et al., 2022). Additionally, in 2021, Voabil et al. constructed a patient-derived tumor fragment platform from fresh breast cancer biopsies and found that the presence and size of tertiary lymphoid structures and B cell abundance correlated with the immune activation capacity after PD-1 blockade (Voabil et al., 2021). In 2024, Maulana et al. constructed a tumor-on-chip model with breast organoids co-cultured adjacent to an endothelialized microchannel with constant CAR-T cell perfusion to assess patient-specific CAR-T cell efficacy (Maulana et al., 2024).

In summary, BCOs have transcended the limitations of simple “tumor cell models”, demonstrating broad potential in drug screening, immunotherapy prediction, microenvironment reconstruction, and gene editing. On one hand, their clinical concordance supports “*in vitro* first, *in vivo* later” personalized treatment. On the other hand, 3D and microfluidic platforms co-cultured with immune cells, CAFs, or endothelial cells enable the dynamic intervention of “tumor-microenvironment” interactions, moving beyond static observation to active modulation. With advances in standardized protocols, multi-omics real-time monitoring, and AI-driven analysis technologies, BCOs are poised to become routine clinical tools bridging precision medicine and translational research.

## 6 Challenges and future prospects

Despite substantial progress, mammary organoids and BCOs remain limited by incomplete biological fidelity, insufficient reproducibility, and limited clinical validation. Current models do not fully reproduce vascular perfusion, immune–stromal interactions, mechanical cues, or spatial drug gradients, while variations in tissue processing, matrices, culture media, and passage number hinder cross-study comparison. Future development should therefore focus on models that are biologically relevant, technically reproducible, quantitatively measurable, and clinically testable.

### 6.1 Improving biological fidelity and dynamic modeling

Conventional epithelial organoids generally lack stable vascular, immune, and stromal compartments, whereas static cultures cannot adequately model perfusion, immune-cell trafficking, or treatment-induced microenvironmental changes. Multicellular co-culture, vascularized models, and organoid-on-a-chip platforms may partially address these limitations. Future mammary organoids should improve hormonal responsiveness and functional maturation, whereas BCOs should better preserve clinically relevant tumor–microenvironment interactions. However, increasing complexity may reduce reproducibility and experimental clarity. Model composition should therefore be selected according to the intended application rather than pursuing maximal complexity.

### 6.2 Standardized quality control and organoid biobanks

Standardization should cover tissue acquisition, sample processing, culture conditions, passaging, cryopreservation, molecular characterization, and functional analysis. Recently published guidance and a breast cancer-specific consensus standard provide initial frameworks for organoid manufacturing and quality assessment (Ahn et al., 2024; Chen et al., 2026). Quality control should include sample authentication, contamination testing, comparison with parental tissues, and monitoring of passage-dependent changes. Particular attention should be paid to normal epithelial-cell overgrowth and the loss of clinically relevant molecular features during culture (Schwerd-Kleine et al., 2025).

Future biobanks should extend beyond collections of expandable lines toward longitudinal and clinically annotated resources. Existing BCO biobanks preserve important features across breast cancer subtypes and patient populations. Incorporating matched primary and metastatic tumors, pre- and post-treatment samples, molecular profiles, drug responses, and clinical outcomes would improve their value for studying tumor evolution, treatment resistance, and biomarker validation.

### 6.3 Digital pathology and patient-avatar platforms

Digital pathology, high-content imaging, and artificial intelligence may transform organoid analysis from subjective morphological assessment into quantitative phenotyping. Computational platforms can automatically track organoids and measure morphological and cellular features in two- and three-dimensional datasets (Matthews et al., 2022; Ong et al., 2025). AI-assisted imaging across clinical breast tissues, animal models, and organoid-based systems further illustrates the potential integration of digital pathology with organoid research (Sun et al., 2026). However, differences in imaging systems, culture conditions, and patient populations require harmonized protocols and external validation.

These technologies may also support patient-avatar platforms. A patient avatar should not refer simply to a single PDO, but to a longitudinal framework integrating serial organoids with pathology, molecular profiling, treatment history, and clinical outcomes. Matched pre- and post-treatment BCOs have illustrated the feasibility of modeling patient-specific tumor evolution (Mazzucchelli et al., 2024), while integrated PDO and iPSC-based systems may support assessment of drug efficacy and toxicity (Chitrangi et al., 2023). Nevertheless, organoid avatars cannot fully reproduce systemic pharmacokinetics, host immunity, endocrine regulation, or treatment-associated toxicity and should therefore be considered complementary clinical tools.

### 6.4 Roadmap toward clinical translation

Clinical translation requires analytical validity, clinical validity, and clinical utility. Although several studies have reported concordance between BCO drug responses and patient outcomes, concordance alone does not demonstrate that organoid-guided treatment improves clinical benefit. Prospective studies should use standardized workflows, predefined response criteria, and clinically relevant endpoints while reporting establishment success, assay failure, turnaround time, and predictive performance.

A randomized phase II clinical trial, ORIENTA (NCT06102824), is evaluating whether treatment selection based on patient-derived organoid drug sensitivity testing provides greater clinical benefit than physician-selected treatment in previously treated HER2-negative advanced breast cancer (Wang, 2024). Until prospective results are available, the clinical utility of organoid-guided treatment remains to be established. Automation and assay miniaturization may reduce turnaround time, but routine implementation will also require validated operating procedures, regulatory oversight, laboratory accreditation, and data-governance frameworks (Ahn et al., 2024; Chen et al., 2026).

Collectively, future progress will depend not simply on generating more complex organoids, but on improving biological relevance, standardization, quantitative analysis, and prospective clinical validation. Integration with dynamic culture systems, quality-controlled biobanks, digital pathology, and patient-avatar platforms may progressively expand the utility of mammary organoids and BCOs in mechanistic research and precision oncology (Table 8).

**Table 8:** List of abbreviations.

Abbreviation	Definition
BCOs	Breast cancer organoids
CAFs	Cancer-associated fibroblasts
CDOs	Circulating tumor cell-derived organoids
CTC	Circulating tumor cell
ECM	Extracellular matrix
EDCs	Endocrine-disrupting chemicals
EHS	Engelbreth-Holm-Swarm
ESCs	Embryonic stem cells
HER2^+^	HER2-positive
hiPSCs	Human induced pluripotent stem cells
iPSCs	Induced pluripotent stem cells
MaSCs	Mammary stem cells
MEMOs	Multi-lineage ESC-derived mammary organoids
mESCs	Mouse embryonic stem cells
MGO	Methylglyoxal
OoC	Organoid-on-a-chip
PBMC	Peripheral blood mononuclear cell
PDMS	Polydimethylsiloxane
PDOs	Patient-derived tumor organoids
PDOTS/MDOTS	Patient/murine derived organotypic tumor spheroids
PSCs	Pluripotent stem cells
TAMs	Tumor-associated macrophages
TME	Tumor microenvironment
TNBC	Triple-negative breast cancer

## CRediT authorship contribution statement

**Yuan Meng:** Writing – original draft. **Pinyang Bai:** Writing – original draft. **Lingxian Zhang:** Writing – review & editing. **Mengna Zhang:** Writing – review & editing, Conceptualization. **Cheguo Cai:** Writing – review & editing, Conceptualization.

## Declaration of competing interest

The authors declare that they have no known competing financial interests or personal relationships that could have appeared to influence the work reported in this paper.
